# Clinically Important Decrease in Liver Stiffness Following Treatment for Hepatitis C: Outcome of the TraP HepC Nationwide Elimination Program

**DOI:** 10.3390/jcm14113982

**Published:** 2025-06-05

**Authors:** Smári Freyr Kristjánsson, Sigurdur Olafsson, Magnús Gottfredsson, Thorvardur Jon Love, Einar Stefán Björnsson

**Affiliations:** 1 Faculty of Medicine, University of Iceland, 101 Reykjavik, Iceland; sfk1@hi.is (S.F.K.); magnusgo@landspitali.is (M.G.); thorvard@landspitali.is (T.J.L.); 2 Division of Gastroenterology and Hepatology, Landspitali—The National University Hospital of Iceland, 101 Reykjavik, Iceland; sigurdol@landspitali.is; 3 Department of Infectious Diseases, Landspitali—The National University Hospital of Iceland, 101 Reykjavik, Iceland; 4 Department of Science, Landspitali—The National University Hospital of Iceland, 101 Reykjavik, Iceland

**Keywords:** chronic hepatitis C, hepatitis C virus, liver stiffness, liver cirrhosis

## Abstract

**Background/Objectives:** Direct-acting antiviral (DAA) therapy has been highly successful in treating chronic hepatitis C (CHC). The nationwide Treatment as Prevention of Hepatitis C (TraP HepC) initiative that was launched in Iceland in 2016 utilized liver stiffness measurements (LSM) to assess liver fibrosis at baseline and follow-up. We aimed to determine changes in liver stiffness among patients following treatment with DAAs and evaluate risk factors associated with hepatic fibrosis. **Methods:** Eligible CHC patients with liver stiffness of >9.5 kilopascals (kPa) before DAA treatment were invited for a follow-up visit in 2024. Risk factors for cirrhosis were registered, LSM performed, and liver enzymes, blood lipids, and glucose levels measured. Changes in liver stiffness were compared to baseline measurements, and correlations with risk factors were analyzed. **Results:** A total of 96 patients had LSMs > 9.5 kPa at treatment initiation. During the follow-up period, 61 were eligible for participation, 38 consented, and 34 (35%) died. The total follow-up was 258.3 person-years. The median follow-up period between measurements was 7.1 years. The median liver stiffness decreased from 17.2 kPa to 7.3 kPa (*p* < 0.01), and 80% of those with cirrhosis (>12.5 kPa) regressed to non-cirrhotic values. High BMI and daily alcohol consumption were significantly associated with increased liver stiffness in 8% of patients. **Conclusions:** In this single-arm, pre-post pilot study, liver stiffness regressed significantly in 92% of patients who were cured of CHC. Patients with other persistent risk factors following cure, such as obesity and alcohol abuse, were the only patients who had increased liver stiffness at the end of follow-up.

## 1. Introduction

The advent of Direct-Acting Antivirals (DAAs) in 2011 marked a significant breakthrough in the management of infections caused by the hepatitis C virus (HCV) [1,2]. DAAs provide potent antiviral inhibition with higher cure rates, reduced treatment duration, and fewer side effects than previous therapies against HCV [2].

The TraP HepC program was launched in January 2016 [3,4]. It had two key aims: (A) to offer a cure to patients and thus reduce the long-term sequelae of chronic hepatitis C (CHC), and (B) to reduce domestic incidence of HCV in the population by 80% prior to the WHO (World Health Organization) goal of HCV elimination by the year 2030 [3,4].

As a part of the management protocol of TraP HepC, and prior to the start of DAAs, liver stiffness measurements (LSMs) were undertaken at baseline by transient elastography (TE) using a FibroScan^®^. Liver stiffness is a marker of liver fibrosis and can identify cases of liver cirrhosis that have not been diagnosed using clinical parameters. The risk of liver-related outcomes, such as cirrhosis, complications of cirrhosis, and hepatocellular carcinoma (HCC), is related to the severity of liver fibrosis in HCV and other chronic liver disorders. LSM results have been shown to predict outcomes in patients with liver disease [5]. There is accumulating evidence that treatment of CHC leads to improvement in liver fibrosis [6,7,8]. In a study from Barcelona, focusing on invasive hepatic hemodynamic measurements, LSM was also undertaken in patients achieving HCV cure by means of antiviral therapy [7]. Liver stiffness decreased markedly after sustained virological response (SVR). After at least three years following HCV cure, fibrosis had improved in 57% of patients; fibrosis progression was seen among 7%, and there was no change in 36% of patients [7]. In these studies [6,7,8], other risk factors for the development of liver fibrosis, such as the overconsumption of alcohol and features of metabolic syndrome, were not analyzed. In the current study, patients participating in TraP HepC were invited to undergo follow-up LSM measurements in 2024 in order to evaluate potential changes in liver stiffness following cure.

The aims of the current study were to investigate the impact of antiviral therapy on the development of liver fibrosis and also to assess the contribution of other risk factors in the setting of a population-based treatment initiative for CHC. This analysis was designed as a single-arm, pre-post pilot study to evaluate longitudinal changes in fibrosis and to confirm trends observed in prior real-world cohorts.

## 2. Materials and Methods

### 2.1. Setting and Collection of Baseline Data

All patients eligible for the current study had participated in the nationwide TraP HepC initiative launched in Iceland in 2016 from 2016 to 2019. The study protocol and the management have previously been described in detail [3]. Briefly, all patients with HCV infection in Iceland, based on positive PCR testing, were offered DAA treatment free of charge, and no restrictions were placed by stage of liver fibrosis or abstinence from drug or alcohol use. Laboratory parameters such as full blood count, electrolytes, creatinine, and liver tests: ALT, AST, GGT, ALP, and bilirubin, were measured at baseline, prior to starting the DAA treatment. Comorbidities were assessed using the Charlson Comorbidity Index [9]. Participants underwent LSM measurement using a FibroScan^®^ (Echosens, Paris, France) prior to initiation of the DAA therapy, and their baseline value in kPa was registered.

### 2.2. Follow-Up Study

Inclusion criteria for participation in the current study were moderate to advanced fibrosis at baseline, with stage III–IV fibrosis, according to LSM (>9.5 kPa). Exclusion criteria included the presence of hepatocellular carcinoma (HCC) or decompensated cirrhosis, along with other complications that would impair participants’ ability to engage in the study, such as neurological disorders or reduced mobility.

Participants were contacted by telephone and briefed on the nature and aims of the study. Additional information was sent via e-mail, appointments were scheduled for those who agreed to participate, and patients signed informed consent.

After fasting overnight, the participants underwent LSM using a FibroScan^®^. The participants completed a standardized questionnaire regarding alcohol consumption, hypertension, sleep apnea, history and treatment for diabetes or lipid-lowering treatment, and change in weight since the baseline LSM. Before participation, information was obtained on the development of HCC and/or hepatic decompensation.

Risk factors influencing liver stiffness were selected with an emphasis on known risk factors. This included age, sex, waist circumference, diabetes mellitus, obesity, sleep apnea, liver stiffness at baseline, and alcohol consumption, with established risk thresholds at 30 g/day for men and 20 g/day for women [10]. Height and weight were also measured in order to calculate the body mass index (BMI), along with waist circumference and blood pressure.

Additionally, histories of obesity and type II diabetes were obtained. Fasting blood sugar was measured, as well as plasma triglycerides, plasma cholesterol, and high-density lipoprotein (HDL) cholesterol. Information was obtained about the causes of death among those who had died during follow-up. STROBE guidelines were followed (Supplementary Table S1).

### 2.3. Liver Stiffness Measurements

One of the authors (SFK) performed all the LSM measurements after completing a training course and passing a certification test in FibroScan^®^ measurements conducted by Echosens. At least 10 valid measurements had to be taken, with invalid measurements comprising less than 40% at the same position. The interquartile range (IQR) of the measurement results needed to be less than 30% of the median value.

If these criteria were not fulfilled, the measurements were repeated. In order to separately analyze patients with cirrhosis (>12.5 kPa), these were separately compared with those without cirrhosis.

### 2.4. Statistical Analysis

Microsoft Excel 2010 (Microsoft, Redmond, WA, USA) and R 4.0.2 (R Foundation for Statistical Computing, Vienna, Austria. URL: https://www.R-project.org/) were used for data analysis. A description of the data is provided by means, medians, range, and interquartile range (IQR) or counts and percentages. The Kruskal–Wallis test was used to compare groups with continuous variables, and Fisher’s exact test for dichotomous variables. All statistical tests were two-tailed, and the level of significance was set at 0.05.

### 2.5. Ethics Approval

The study was approved by the ethical committee of the Landspitali University Hospital (LSH-66-2023).

## 3. Results

**Figure 1:**
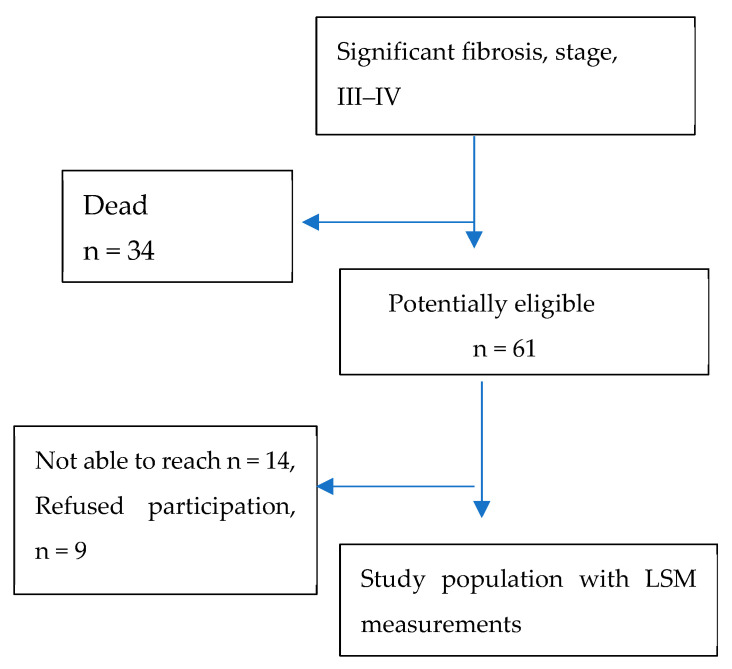
Flow chart of the study cohort and the reasons for exclusions.

At the launch of TraP HepC in 2016 until 2019, 705 patients had confirmed HCV infection with at least one positive HCV PCR test. Of these 705 patients, 692 (98.2%) had undergone LSM prior to initiation of DAA therapy. In total, 95 of 692 (14%) had moderate or advanced fibrosis (>9.5 kPa). At the time of patient recruitment, several patients could not be reached by telephone and/or e-mail or by regular mail or had moved abroad, as detailed in Figure 1. At the time of follow-up, 34 (36%) had died. Of the remaining 61, 38 patients consented (61%), and therefore, we were able to perform LSM on them, yielding a total of 258.3 person-years of follow-up. Among these patients, 82% were males, with a median age of 59 years (IQR 51–65, range 33–79) (Table 1).

**Figure 2:**
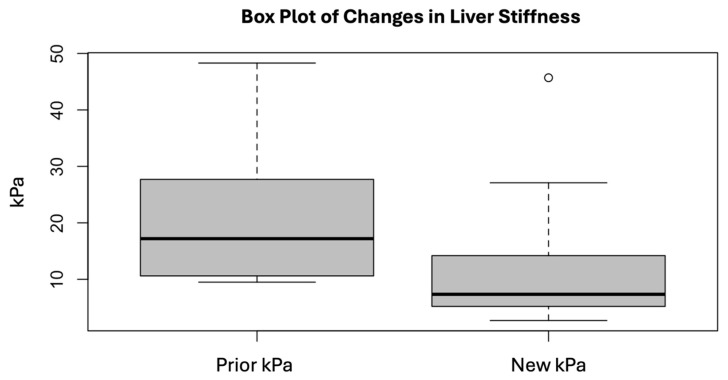
Box plot depicting changes in kPa value over the period. *p* < 0.01. The circle means an outlier.

The median time between the LSM was 7.1 (IQR 6.3–8.0) years. None of these patients were found to have decompensated or developed HCC during the follow-up. Among those who had died, 18/34 (59%) were due to causes that were non-liver related, either related to addiction, accidents, heart and lung problems, or infections. A total of six (18%) were liver-related, five developed HCC, four died of malignancies other than HCC, five had an unknown cause of death, and one died abroad. The median LSM values at baseline and at follow-up in the study cohort (n = 38) are shown in Table 1. The LSM values decreased significantly after successful DAA therapy (Figure 2).

**Figure 3:**
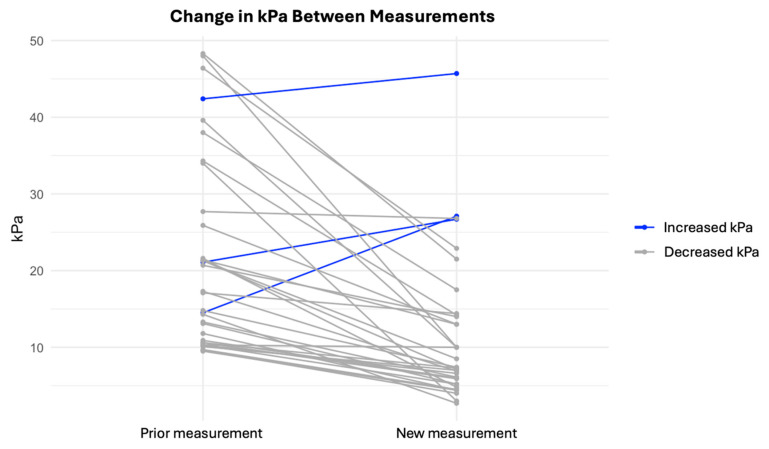
Paired line plot depicting changes in LSM. Decreasing kPa values are shown in grey, and increasing kPa values are shown in blue. *p* < 0.01.

The median before treatment was 17.2 (10.5–26.3) kPa and 7.3 (5.4–14.1) kPa at follow-up (*p* < 0.01). Thus, liver stiffness as measured with the TE declined 42% over a median of seven years following completion of successful DAA therapy. A total of 92% (35/38) of the patients had a lower degree of fibrosis at follow-up, according to the LSMs (Figure 3).

**Figure 4:**
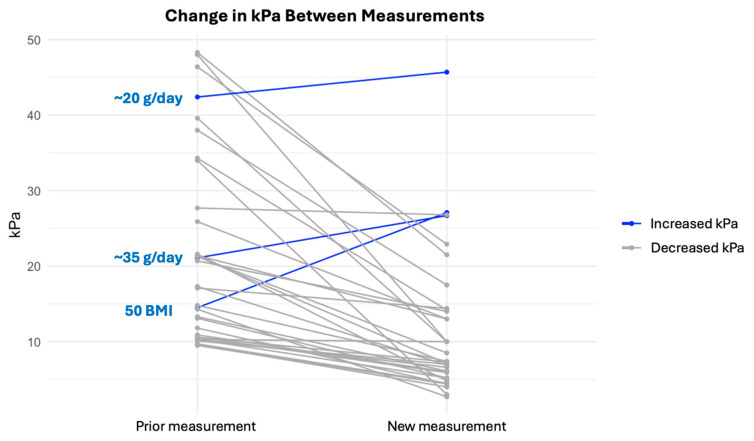
Paired line plot depicting changes in LSM. Decreasing kPa values are shown in grey, and increasing kPa values are shown in blue, showing those who drank at least 20 g or 35 g of alcohol per day, and one patient with morbid obesity and a BMI of 50.

The three patients who had increased LSM values during follow-up in the current study had other risk factors, i.e., overconsumption of alcohol (n = 2) and morbid obesity (BMI = 50) (Figure 4).

**Figure 5:**
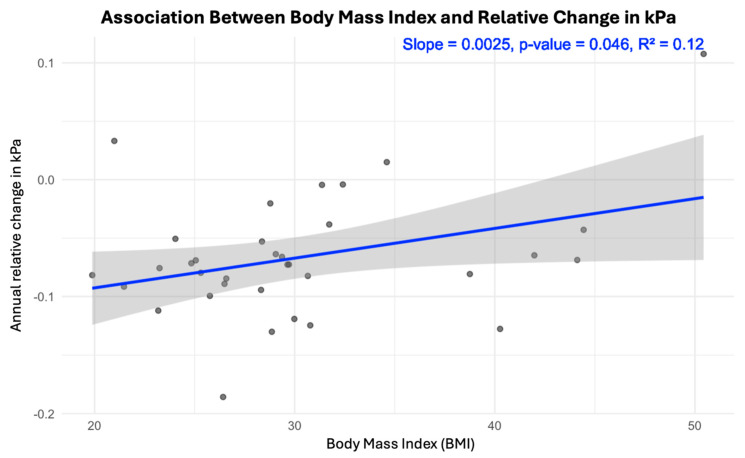
Scatter plot with linear regression depicting the association between BMI and annual relative kPa change. *p* = 0.046. The blue line is a correlation regression line. The grey shaded area around the blue regression line in the plot represents the confidence interval of the regression estimate—a 95% confidence interval.

**Figure 6:**
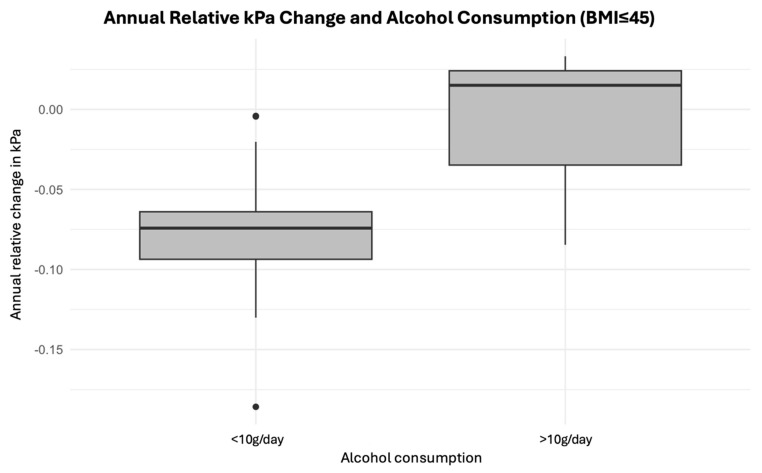
Box plot of annual relative kPa change by alcohol consumption. A BMI cutoff of 45 was applied, as BMI had been shown to influence annual relative kPa change. *p* < 0.05.

Interestingly, a lower BMI was associated with a greater decrease in LSM in kPa at follow-up (*p* = 0.046) (Figure 5). Furthermore, patients who reported daily drinking of more than 10 g of alcohol per day had significantly higher LSM (Figure 6). The presence of hypertension, diabetes mellitus, and sleep apnea was not found to be associated with changes in the LSM measurements.

## 4. Discussion

In this single-arm, pre-post pilot study, without a control group, changes in liver stiffness were investigated in a population-based sample of patients receiving DAA treatment for HCV, demonstrating an important decrease in LSM following HCV cure. After a median of seven years following completion of DAA therapy, 92% of patients with initially high values (>9.5 kPa) had a decrease in fibrosis scores. Importantly, 80% of those with cirrhosis (>12.5 kPa) had regression to non-cirrhotic values. A few patients had an increase in LSM; this was associated with other risk factors for liver disease, such as overconsumption of alcohol or morbid obesity.

Among 96 patients who had moderate to advanced fibrosis at the time of treatment initiation, a total of 36% had died. This was mainly related to non-liver-related mortality, whereas in 18%, the causes were deemed to be liver-related, mainly deaths from HCC. The causes of death were frequently associated with addiction, which is well recognized in patients who have been infected with HCV [11,12,13]. A number of patients could not be reached as they did not answer phone calls or other means of contact, and several were lost to follow-up, as has been the case in other similar studies on fibrosis measurements in HCV patients [14,15].

Nevertheless, it can be argued that the relatively high participation rate (61%) among patients in this population-based sample makes the results more generalizable.

Transient elastography (TE) undertaken in the current study has replaced liver biopsy and is the primary method for staging purposes of liver disease in these patients. TE is a validated methodology for assessing fibrosis in HCV and has been shown to have good reproducibility and diagnostic accuracy [16].

Advanced fibrosis is the leading risk factor for decompensation and HCC in patients with HCV and other chronic liver disorders. In a landmark study from the US, patients with HCV who had been treated with DAAs, who achieved SVR, were found to have a 76% reduction in HCC during long-term follow-up [17]. The results of the current study demonstrated a clinically important reduction in LSM and a decrease in fibrosis scores in 92% of patients. A total of 25 (66%) had evidence of cirrhosis according to the TE measurements, which regressed below 12.5 kPa, which is the cut-off for cirrhosis according to TE, in 20 patients (80%). Similarly, a recent study on the incidence of cirrhosis in Iceland from 2016 to 2022 demonstrated that HCV was the culprit in 15% of patients, and over the study period, the incidence of cirrhosis due to HCV dropped rapidly [18]. During the last two years of the incidence study, only 1–2 cases of HCV cirrhosis were diagnosed annually [18]. This was associated with providing unrestricted access to DAA through the nationwide TraP HepC initiative in Iceland [3,4].

In the present study, patients with higher pre-treatment LSM values at baseline were found to have a more significant decline in liver stiffness after DAA therapy and SVR. This is in agreement with a recent study from Thailand [19]. In that study, with two years of follow-up in 57 patients, pre-treatment values were 15 kPa and declined to 9.6 kPa at a two-year follow-up [19]. In a multivariate analysis, baseline TE values > 9.5 were positively associated with a TE improvement, which is at least 30% [19]. This is similar to the results of our study, where the TE improvement was 42%. However, the even greater improvements presented here could be related to a longer follow-up of a median of seven years. A better outcome among patients with higher pre-treatment values has also been demonstrated in a meta-analysis of LSM after DAA therapy, among patients with higher pre-treatment values [20].

Along the same lines, we also found that the vast majority with cirrhosis (80%), according to the TE values, regressed to non-cirrhotic values. This is in line with a study from Germany by Pietch et al., who found that in patients with cirrhosis, fibrosis declined significantly more than in patients without cirrhosis [21]. The study by Pietsch et al. showed a further decline from two years to eight years of follow-up, from 9.3 kPa to 7.9 kPa [21]. These results are remarkably similar to the results of the current study and are probably related to the longer follow-up in patients with SVR. A decrease in liver stiffness after HCV eradication has also been reported in other somewhat heterogeneous studies [22,23,24,25].

Most other previous studies have had shorter follow-ups, such as two [19], three [14], four [15], and five years [26]. In a recent study from Norway, the median liver stiffness was found to be 6.3 kPa after a four-year follow-up from a baseline value of 8.1 [15]. The somewhat lower values at the end of follow-up might be due to a younger median age in the Norwegian cohort, 49 years vs. 58 years in the current study; furthermore, the patients in Norway were not pre-selected by moderate or advanced fibrosis as in the current study.

Our results demonstrated that fibrosis is reversible and that DAA therapy is associated with a clinically important reduced risk of the development of decompensated liver cirrhosis and HCC development, although it was not the aim of the current study to analyze this. The reversibility of fibrosis and cirrhosis has also been shown in other studies after DAA therapy, leading to SVR [27,28].

Three patients in the present study had a worsening of fibrosis, and in all cases, other risk factors were present, including alcohol abuse and morbid obesity. Thus, it seems important to follow these patients carefully after treatment and try to modify their other risk factors for liver disease.

The present study has several strengths. It is population-based and, therefore, all patients with moderate to advanced fibrosis in Iceland who received DAA therapy were potentially eligible. Apart from the evaluation of liver stiffness, other risk factors for liver disease were carefully evaluated, and clinical follow-up of these patients was thorough. The study also has some limitations. The study cohort was relatively limited in size, and a considerable number of patients had died during follow-up. This allows for several biases. For example, attrition bias—not only due to the 34 participants who died, but also because 23 (24%) could not be reached or declined participation, potentially skewing the results. Selection bias cannot be excluded if those who participated in follow-up differed systematically from those who did not. Additionally, several potential sources of bias and confounding were not addressed. Important factors, such as continued illicit drug use, mental health status, and medication adherence, were not assessed, though they may have influenced liver stiffness outcomes. Furthermore, key risk factors such as alcohol use and weight could have varied significantly over the seven-year follow-up period; however, they were not measured longitudinally. Lastly, the absence of a control group limits the ability to attribute observed improvements solely to the intervention.

## 5. Conclusions

Overall, 92% of patients were found to have a decline in fibrosis scores after a median of seven years of follow-up. A total of 80% of patients with cirrhosis, according to LSM, regressed to non-cirrhotic values. Fibrosis scores worsened only in patients with important risk factors for liver disease, such as alcohol abuse and obesity.

## Figures and Tables

**Table 1:** Demographics, LSMs at baseline and at follow-up, clinical variables, and laboratory parameters at follow-up.

	HCV Patients n = 38
Age, median, IQR,	58 (55–65),
Gender, males (%)	31 (82%)
Time interval between measurements (years)	7.2 (6.4–17.9)
LSM at baseline (kPa)	17.2 (10.5–26.3)
LSM at follow-up (kPa)	7.3 (5.4–14.1)
LSM decrease,	35/38 (92%)
LSM increase	3/38 (8%)
BMI	29 (25–32)
Waist circumference	101 (93–112)
Alcohol use g/day	0 (0–1.4)
Hypertension, n (%)	21(38%)
Diabetes mellitus, n (%)	6 (16%)
Sleep apnea	6 (16%)
ALT (U/L)	25 (16–34)
AST (U/L)	26 (21–31)
ALP (IU/L)	80 (66–95)
GGT (U/L)	39 (23–55)
Bilirubin umol/L	12 (8–16)
INR	1.0 (0.9–1.1)
Platelets	243 (175–311)
Blood sugar, fasting	5.9 (5.5–6.3)
HbA1c	35 (31–40)
Cholesterol	4.7 (4–5.4)
HDL	1.3 (1.1–1.5)
Triglycerides	1.2 (0.8–1.5)

## Data Availability

Data availability is available upon request.

## Supplementary Materials

The following supporting information can be downloaded at: https://www.mdpi.com/article/10.3390/jcm14113982/s1, Table S1: STROBE Statement—Checklist of items that should be included in reports of cohort studies.

## Abbreviations

The following abbreviations are used in this manuscript: Direct-Acting Antivirals (DAAs), hepatitis C virus (HCV), chronic hepatitis C (CHC), transient elastography (TE), WHO (world health organization), hepatocellular carcinoma (HCC), liver stiffness measurements (LSMs), sustained virological response (SVR), body mass index (BMI), high density lipoprotein (HDL), kilopascals (kPa).
